# Osteoarthritis and risk of type 2 diabetes: A two‐sample Mendelian randomization analysis

**DOI:** 10.1111/1753-0407.13451

**Published:** 2023-07-31

**Authors:** Xing Xing, Yining Wang, Faming Pan, Guoqi Cai

**Affiliations:** ^1^ Department of Epidemiology and Biostatistics, School of Public Health Anhui Medical University Hefei China; ^2^ The Inflammation and Immune Mediated Diseases Laboratory of Anhui Province Anhui Medical University Hefei China; ^3^ Menzies Institute for Medical Research, University of Tasmania Hobart Australia

**Keywords:** Mendelian randomization, metabolites, osteoarthritis, type 2 diabetes, 骨关节炎, 2型糖尿病, 代谢物, 孟德尔随机化

## Abstract

**Background:**

Physical inactivity is an independent risk factor for type 2 diabetes (T2D). Osteoarthritis (OA) is a common joint disease that limits patients' physical activity, which may increase risk of other chronic diseases including T2D. However, studies evaluating the effect of OA on T2D are scarce. This study aimed to investigate the causal effect of knee and hip OA on risk of T2D from a genetic perspective.

**Methods:**

We performed two‐sample Mendelian randomization (MR) analyses to obtain nonconfounding estimates of the effect of OA on T2D risk. Single nucleotide polymorphisms (SNPs) from genome‐wide association studies were selected as genetic instruments for radiographic knee and hip OA (ie, Kellgren–Lawrence grade ≥2). The associations of these SNPs with T2D were evaluated in participants from the UK Biobank. Sensitivity analyses were conducted to test the robustness of the MR results.

**Results:**

Genetic predisposition of knee but not hip OA was significantly associated with an increased risk of T2D (knee OA: odds ratio [OR] 1.18, 95% confidence interval (CI) 1.09–1.27, *p* <.001; hip OA: OR 1.04, 95% CI 0.94–1.16, *p* = .425). Sensitivity analyses showed that the main findings are robust.

**Conclusion:**

The current study provides genetic evidence supporting that knee OA is a potential risk factor for T2D.

## Graphical abstract

**Highlights**

- We used data from the genome‐wide association studies database and two‐sample mendelian randomization method, we found that genetic evidence supporting that knee osteoarthritis is a potential risk factor for type 2 diabetes.

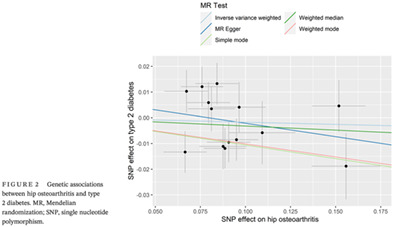

## 摘要

背景:缺乏运动是2型糖尿病(T2D)的独立危险因素。骨关节炎(OA)是一种常见的关节疾病, 限制了患者的运动能力, 可能增加其他慢性疾病(包括T2D)的风险。然而, 评估OA对T2D影响的研究很少。本研究旨在从遗传角度探讨膝关节和髋关节OA对T2D风险的因果效应。

方法:我们进行了双样本孟德尔随机化(MR)分析, 以获得OA对T2D风险无偏估计的影响。我们从全基因组关联研究(GWAS)中选择了单核苷酸多态性(SNPs)作为放射学膝关节和髋关节OA(即Kellgren‐Lawrence分级≥2)的遗传工具。然后评估这些SNPs与参与者来自英国生物库的T2D的关联性。进行敏感性分析以测试MR结果的稳健性。

结果:膝关节OA而非髋关节OA的遗传易感性显著增加T2D的风险(膝关节OA:优势比(OR)1.18, 95%置信区间(CI)1.09至1.27, p < 0.001, 髋关节OA:OR 1.04, 95% CI 0.94至1.16, p=0.425)。敏感性分析显示主要发现是稳健的。

结论:当前研究提供了遗传证据支持膝关节OA是T2D的潜在危险因素。

## 1 INTRODUCTION

Type 2 diabetes (T2D) is one of the leading noncommunicable diseases globally. In 2021, approximately 537 million adults (20–79 years) are living with diabetes, of whom 90% had T2D,^1^ and diabetes caused at least $966 billion in health expenditure.^1^ Physical inactivity is an independent risk factor for T2D.^2^ Osteoarthritis (OA) is the most common joint disease and the leading cause of disability.^3^ Although OA and T2D are common chronic diseases that often coexist,^4^, ^5^ little is known about the role of OA in the risk of T2D. Recognizing the causal relationship between OA and T2D has important clinical implications for disease management and drug development.

Epidemiological studies evaluating the association between OA and diabetes have inconsistent results. In two meta‐analyses of observational studies with different designs (eg, cross‐sectional, case–control, and cohort), patients with OA at different sites were more likely to have T2D than those without.^5^, ^6^ Three cohort studies have assessed the effect of OA on risk of T2D. Kendzerska et al^7^ found that the presence of hip/knee osteoarthritis was an independent predictor of the development of T2D. Rahman et al^8^ reported that young adults and older women with OA had a higher risk of developing diabetes compared to non‐OA patients of the same age. However, Frey et al^9^ found no significant association between clinical OA and T2D. Observational studies may be biased by residual confounding; therefore, it is uncertain whether OA would lead to an increased risk of T2D. Genome‐wide association study (GWAS) has significantly contributed to identifying genetic variations associated with common complex diseases,^10^ which provides insights into the genetic basis of many complex traits.^11^ Mendelian randomization (MR) analysis uses genetic variation as an instrumental variable (IV) to test for the causal association between risk factors and diseases.^12^ Because genotypes precede disease progression and are mainly independent of postnatal lifestyle or environmental factors, this technique can minimize confounding and avoid reverse causality bias.^13^ In this study, we aimed to evaluate the causal effects of knee and hip OA (KOA and HOA) on the risk of T2D using a two‐sample MR approach.

## 2 METHODS

### 2.1 Study design

This genetic association study was conducted according to the Strengthening the Reporting of Observational Studies in epidemiology using Mendelian Randomization (STROBE‐MR).^14^ We used publicly available summary data released by GWAS studies of KOA, HOA, and T2D. These studies have been approved by corresponding institutional review boards, and all participants provided informed consent.

#### 2.1.1 Genetic instruments for OA

The summary‐level data for KOA and HOA were obtained from a genome‐wide meta‐analysis conducted by the UK Biobank and the Arthritis Research UK Osteoarthritis Genetics (arcOGEN) resources, which included 24 955 cases with KOA, 15704 cases with HOA, and 378 169 controls of European descent.^15^ The arcOGEN samples were collected based on clinical evidence of disease to a level requiring joint replacement or radiographic OA (Kellgren–Lawrence grade 2).^15^ Detailed data and statistics about the samples, genotyping, and imputation have been introduced previously.^15^ We chose SNPs as IVs for all exposures (KOA and HOA, respectively) from the IEU Open GWAS database, a database of genetic associations from GWAS summary data sets (https://gwas.mrcieu.ac.uk/).^16^ In GWAS of OA, SNPs that reached genome‐wide significance (*p* < 5 × 10^−8^) were used as IVs for KOA and HOA, respectively. We checked SNPs for independent inheritance (<0.001) without linkage disequilibrium with each other and several bases between two SNPs (kb >10 000). Then, we selected the reference sample formed by the European ancestral individuals from the 1000 genomes project to estimate the allele frequency and linkage disequilibrium level.^17^ We also calculated the F‐statistic of SNPs to determine the strength of the instruments; an F‐statistic >10 indicates a low bias due to sample overlap.^18^, ^19^ Detailed information about these IVs is shown in Supplementary File S1.

### 2.2 Genetic associations of SNPs for T2D

Publicly available summary‐level data on genetic variations associated with T2D were obtained from a large meta‐analysis of GWAS (62 892 cases and 596 424 controls of European ancestry).^20^ The profile of this GWAS is provided in Supplementary File S1.

### 2.3 Statistical analysis

We conducted two separate two‐sample MR analyses to test the potential causal associations between KOA/HOA with the risk of T2D. Inverse variance weighted (IVW) method was used as the primary analysis for the causal association of KOA/HOA with T2D risk. IVW can provide more precise estimates when all IVs are valid.^21^ MR‐Egger, weighted median, simple mode, and weighted mode methods^18^, ^21^, ^22^ were also conducted. A *p* value <.05 represented a statistically significant causal association of KOA/HOA with T2D. The multiplicative random‐effect IVW method was used when the number of IVs is >3, otherwise the fixed‐effect IVW method was used.^23^ Statistical power was calculated using the method described by Brion et al.^24^

Some sensitivity analyses were conducted. First, both Mendelian Randomization Pleiotropy RESidual Sum and Outlier (MR‐PRESSO) and MR‐Egger^22^, ^25^ were used to test the pleiotropy of genetic instruments for OA in the T2D GWAS dataset. MR‐PRESSO was used in order to identify horizontal pleiotropic outliers in multi‐instrument summary‐level MR testing. MR‐PRESSO identifies horizontal pleiotropic outlier variants and provides an outlier‐corrected estimate.^22^ Heterogeneity across IVs is also an indicator of pleiotropy. Cochran's Q test and MR‐Egger regression in the IVW method^26^, ^27^ were used to test the heterogeneity of genetic instruments for OA in the T2D GWAS data set. A *p* value >.05 represented no statistically significant pleiotropy and heterogeneity. Second, the leave‐one‐out sensitivity analysis was used to evaluate the effect of each IV on the risk of T2D. Third, we searched online webtool Phenoscanner to determine pleiotropic SNPs that were also significantly (*p* = 5E‐08) associated with other risk factors (confounders) of T2D (Supplementary Table S5). Considering that high body mass index (BMI) is an important risk factor for T2D,^28^ we conducted a sensitivity analysis by removing SNPs that were associated with BMI.

All statistical analyses were performed using the “TwoSampleMR” package for R version 3.6.1.

## 3 RESULTS

A total of 21 independent SNPs associated with T2D were identified (Supplementary file S1). Five and sixteen SNPs were identified as IVs for KOA and HOA, respectively. All F‐statistics for the IVs associated with KOA and HOA were >10, suggesting all IVs were valid (Supplementary Table S1 and Table 2). The statistical power to detect an odds ratio (OR) of ≥1.50 ranged from 83% to 100% (Supplementary Table S4).

### 3.1 The causality between KOA and T2D

**FIGURE 1:**
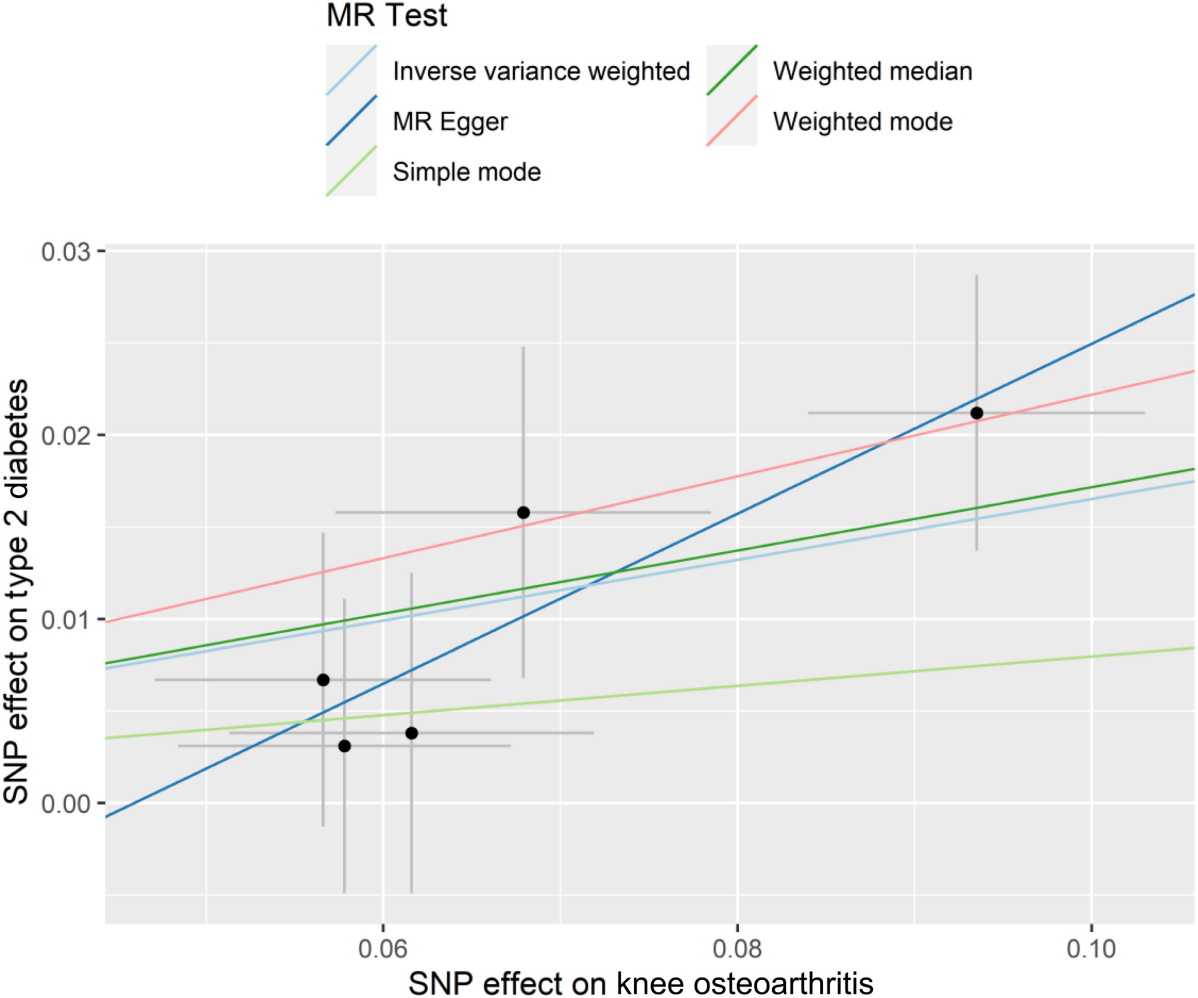
Genetic associations between knee osteoarthritis and type 2 diabetes. MR, Mendelian randomization; SNP, single nucleotide polymorphism.

The results of IVW showed a causal relationship between KOA and an increased risk of T2D (OR = 1.18, 95% confidence interval [CI] 1.09–1.27, *p* <.001) (Table 1 and Figure 1). Results from the weighted median and weighted mode, but not the simple model and MR‐Egger, were consistent with the results of IVW (Table 1).

**TABLE 1:** Two‐sample Mendelian randomized analyses for the associations of knee and hip osteoarthritis with the risk of type 2 diabetes.

Exposure‐outcome	No. of SNP	Methods	OR (95%CI)	*p* value
**KOA‐T2D**	5	MR Egger	1.59 (0.97–2.60)	.164
		Weighted median	**1.19 (1.04–1.35)**	**.009**
		Inverse variance weighted	**1.18 (1.09–1.27)**	**<.001**
		Simple mode	1.08 (0.90–1.30)	.436
		Weighted mode	**1.25 (1.07–1.46)**	**.050**
**HOA‐T2D**	16	MR Egger	0.81 (0.51–1.29)	.391
		Weighted median	0.99 (0.92–1.07)	.888
		Inverse variance weighted	1.04 (0.94–1.16)	.425
		Simple mode	0.90 (0.77–1.05)	.195
		Weighted mode	0.90 (0.79–1.03)	.156

Abbreviations: CI, confidence interval; HOA, hip osteoarthritis; KOA, knee osteoarthritis; MR, Mendelian randomization; OR, odds ratio; SNPs, single nucleotide polymorphisms; T2D, type 2 diabetes. Bold text indicates statistically significant results (*p* < 0.05).

Pleiotropy test and heterogeneity test showed no evidence of pleiotropy and heterogeneity (all *p* >.05) (Tables 2 and 3). The symmetry of the funnel plot also confirmed the absence of heterogeneity, which showed that the associations of all IVs of KOA with T2D were robust (Supplementary Figure S2A). The leave‐one‐out sensitivity analysis did not materially change the results of MR analyses (Supplementary Figure S1A). No IVs for KOA were found to be associated with BMI.

**TABLE 2:** Horizontal pleiotropy test for the association of knee and hip osteoarthritis with the risk of type 2 diabetes.

Exposure	Outcome	Egger intercept	Intercept *p* value	MR‐PRESSO Global test‐*p* value	Main MR results *p* value
KOA	T2D	−0.021	.315	.111	.014
HOA		0.027	.290	<.001	.586

Abbreviations: HOA, hip osteoarthritis; KOA, knee osteoarthritis; MR, Mendelian randomization; T2D, type 2 diabetes.

**TABLE 3:** Heterogeneity test for the effect of KOA and HOA on the risk of T2D.

Exposure	Outcome	IVW		MR‐Egger	
		Cochran's Q	Q‐ *p* value	Cochran's Q	Q‐ *p* value
KOA	T2D	2.145	0.709	0.699	.873
HOA		18.704	0.133	17.986	.116

Abbreviations: HOA, hip osteoarthritis; IVW, inverse variance weighting; KOA, knee osteoarthritis; MR, Mendelian randomization; T2D, type 2 diabetes.

### 3.2 The causality between HOA and T2D

**FIGURE 2:**
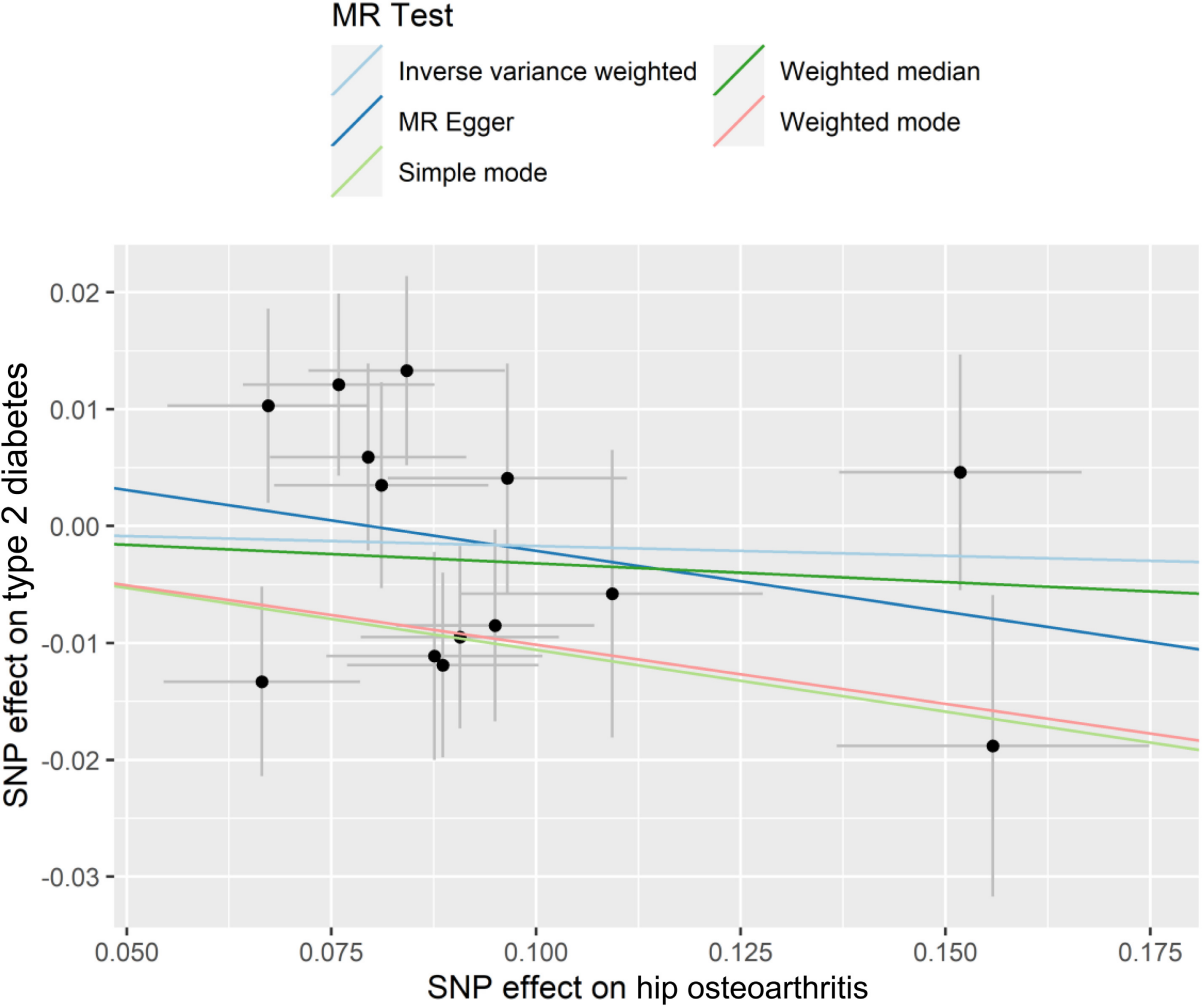
Genetic associations between hip osteoarthritis and type 2 diabetes. MR, Mendelian randomization; SNP, single nucleotide polymorphism.

No statistically significant causal relationships of HOA with risk of T2D were found, based on the IVW, weighted median, MR–Egger regression, weighted mode, and simple mode methods (Table 1). A significant heterogeneity for the association between HOA and risk of T2D was found (Table 2). Therefore, MR‐PRESSO was conducted and showed similar results (Supplementary Table S3). The Cochran's Q test and MR‐Egger regression in the IVW method revealed that the horizontal pleiotropy was unlikely to bias the association between HOA and risk of T2D (Table 3). Results of the slope of the scatter plot, the asymmetry of the funnel plot, and the leave‐one‐out sensitivity analysis showed no causal association between HOA and T2D risk (Figure 2, Supplementary Figure S1B and Supplementary Figure S2B). Two IVs for HOA was found to be associated with BMI (rs3774355 and rs11059094). Sensitivity analysis by removing these IVs did not change the association between HOA and T2D risk (data not shown).

## 4 DISCUSSION

This study was the first to evaluate the causal relationships of KOA and HOA with risk of T2D from a genetic perspective. Using two‐way Mendelian analyses, we found that radiographic KOA but not HOA was a potential risk factor for T2D. The findings of this study suggest an important role of KOA in developing T2D, and the prevention and treatment of KOA may be beneficial for T2D.

Some previous population‐based cohort studies have shown that arthritis, including KOA, was associated with an increased T2D risk.^6^, ^8^ However, OA and T2D are common chronic diseases and share many risk factors (eg, aging, metabolic problems, lack of physical activity, and socioeconomic disadvantage),^29^, ^30^ and some of these factors were not measured or accounted for in these studies. Thus, the observed associations between OA and T2D in population‐based studies may be biased by residual confounding. The present study avoided such confounding effect using instrumental variables and confirmed a detrimental role of KOA in T2D risk.

Although the mechanisms underlying the association between OA and T2D are not well understood, a possible explanation is that functional limitations due to OA led to reduced weight‐bearing activities, increased sedentary behaviors, and weight gain, which are important risk factors for T2D.^31^, ^32^, ^33^, ^34^ Moreover, proinflammatory cytokines, chronic joint inflammation, and endothelial dysfunction in OA may also contribute to developing insulin resistance, dysfunction and destruction of beta cells, and eventually diabetes.^35^, ^36^, ^37^, ^38^, ^39^

We did not find a causal relationship between HOA and T2D risk, suggesting dissimilar effects of KOA and HOA on the development of T2D. Previous studies have shown that metabolic syndrome plays a more significant role in KOA than in HOA.^40^ In addition, genetic factors affecting bone shape are more closely associated with KOA.^41^ Due to the relationship between diabetes prevalence and metabolic syndrome, it is hypothesized that the relationship between diabetes prevalence and KOA is stronger than HOA. Previous studies have indicated that metabolic disorders may contribute to the risk and progression of OA.^42^, ^43^, ^44^ In a systematic review and meta‐analysis,^5^ although OA increased the risk of T2D by 41%, T2D also increased the risk of OA by 46%, even after adjusting for BMI. This combines with our findings suggesting a bidirectional relationship between T2D and OA. However, a recent MR study did not find a significant causal association of T2D and glycemic traits with OA risk.^45^ The inconsistency may be due to the differences in the definition of OA used in observational studies and the MR study. Alternatively, it could also be that the observational findings are biased by reverse causation, as we found in this MR study that KOA had a causal association with T2D risk.

In this study, the strength for the association between OA and risk of T2D may have been underestimated because the definition of OA was based on the Kellgren–Lawrence grades but not clinical symptoms. Previous studies have indicated that patients with radiographic OA do not necessarily have joint symptoms and vice versa,^46^, ^47^ and their physical activity may be less likely to be influenced compared to those with symptomatic OA.^48^ Clinical definitions of OA, such as the American College of Rheumatology criteria, are widely applied in many studies and in clinical practice.^49^, ^50^ In clinical settings, joint symptoms, especially pain, are the primary reason for individuals seeking medical attention.^51^ Therefore, further studies are warranted to understand the role of symptomatic OA on risk of T2D, and we speculated a stronger causal relationship between symptomatic OA and T2D risk.

The strength of this study is that MR analyses provided nonconfounding estimates for the association between OA and T2D and all IVs identified were valid, making our findings more reliable. Our study also has some limitations. First, we cannot exclude the possibility of sample overlap. However, our tests indicated that the impact of sample overlap is small.^18^ Second, the GWAS data were from populations of European ancestry; therefore, the causal relationship between OA and T2D risk should be verified in other populations. Third, heterogeneity was found in the association between HOA and T2D, but the sensitivity analyses showed that our results are robust.

In conclusion, our two‐sample MR analyses showed a significant causal relationship between radiographic KOA but not HOA and an increased risk of T2D.

## AUTHOR CONTRIBUTIONS

Guoqi Cai conceived, initiated, and supervised the project. Xing Xing collected and analyzed the data. Yining Wang and Xing Xing contributed to the interpretation of the results. Xing Xing wrote a draft of the manuscript. All authors critically reviewed and revised the manuscript, and agreed to the published version of the manuscript.

## FUNDING INFORMATION

This work was supported by the National Natural Science Foundation of China (82103933).

## CONFLICT OF INTEREST STATEMENT

The authors declare no conflicts of interest.

## Supporting information


**Figure S1.** The leave‐one‐out plot of the effects of knee osteoarthritis/hip osteoarthritis (KOA/HOA) in type 2 diabetes (T2D).
**Figure S2.** The funnel plot of the effects of knee osteoarthritis/hip osteoarthritis (KOA/HOA) and in type 2 diabetes (T2D).

**Table S1.** Characteristics of SNPs used in Mendelian randomization analysis of the effects of knee osteoarthritis (KOA) in type 2 diabetes (T2D).
**Table S2.** Characteristics of SNPs used in Mendelian randomization analysis of the effects of hip osteoarthritis (HOA) in type 2 diabetes (T2D).
**Table S3.** Two‐sample Mendelian randomized analyses for the associations of hip osteoarthritis with the risk of type 2 diabetes after adjusted by MR‐PRESSO.
**Table S4.** Statistical power for the Mendelian randomization analysis.
**Table S5.** List of genetic variants associated with more than one phenotype.

## Data Availability

Data used in this study are all publicly available. The authors will provide the data upon reasonable request.
