# Supplementary material for: Osteoarthritis and risk of type 2 diabetes: A two‐sample Mendelian randomization analysis
Source: J Diabetes. 2023 Jul 31;15(11):987–93. doi: 10.1111/1753-0407.13451 (PMC10667649; doi:10.1111/1753-0407.13451)
Supplement: Supplementary file 1 — Figure S1. The leave‐one‐out plot of the effects of knee osteoarthritis/hip osteoarthritis (KOA/HOA) in type 2 diabetes (T2D). Figure S2. The funnel plot of the effects of knee osteoarthritis/hip osteoarthritis (KOA/HOA) and in type 2 diabetes (T2D). [file JDB-15-987-s002.docx]

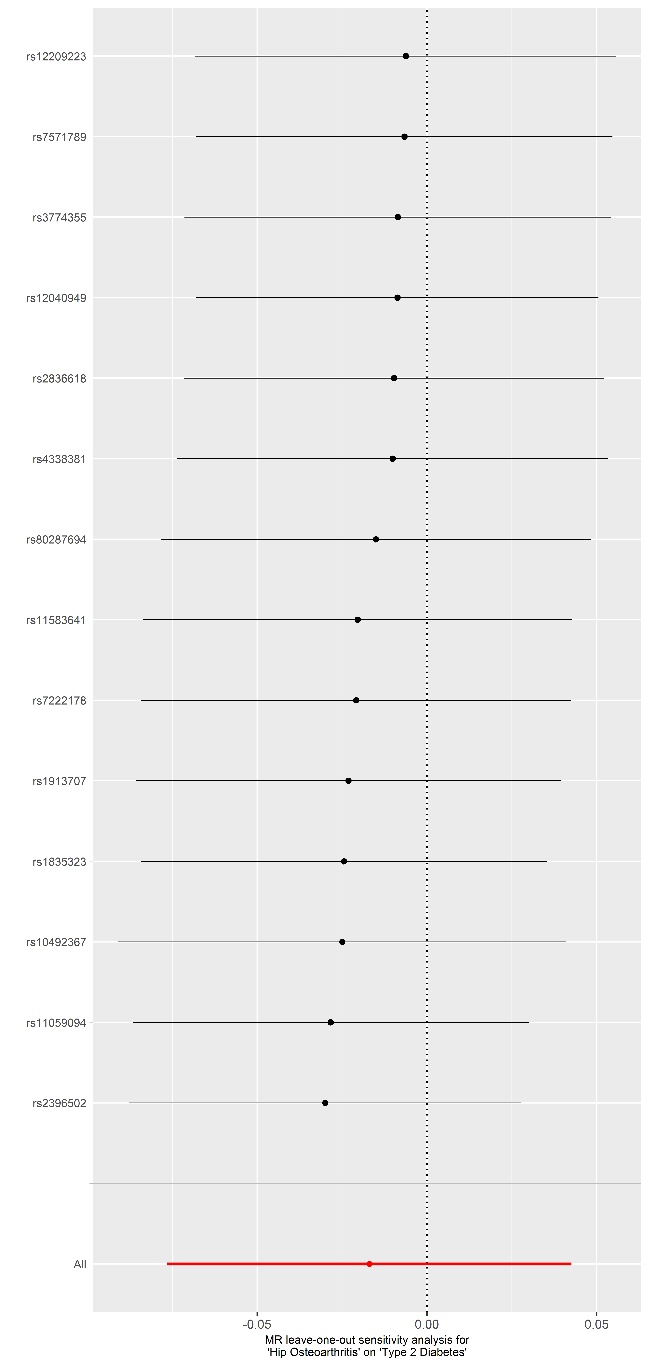

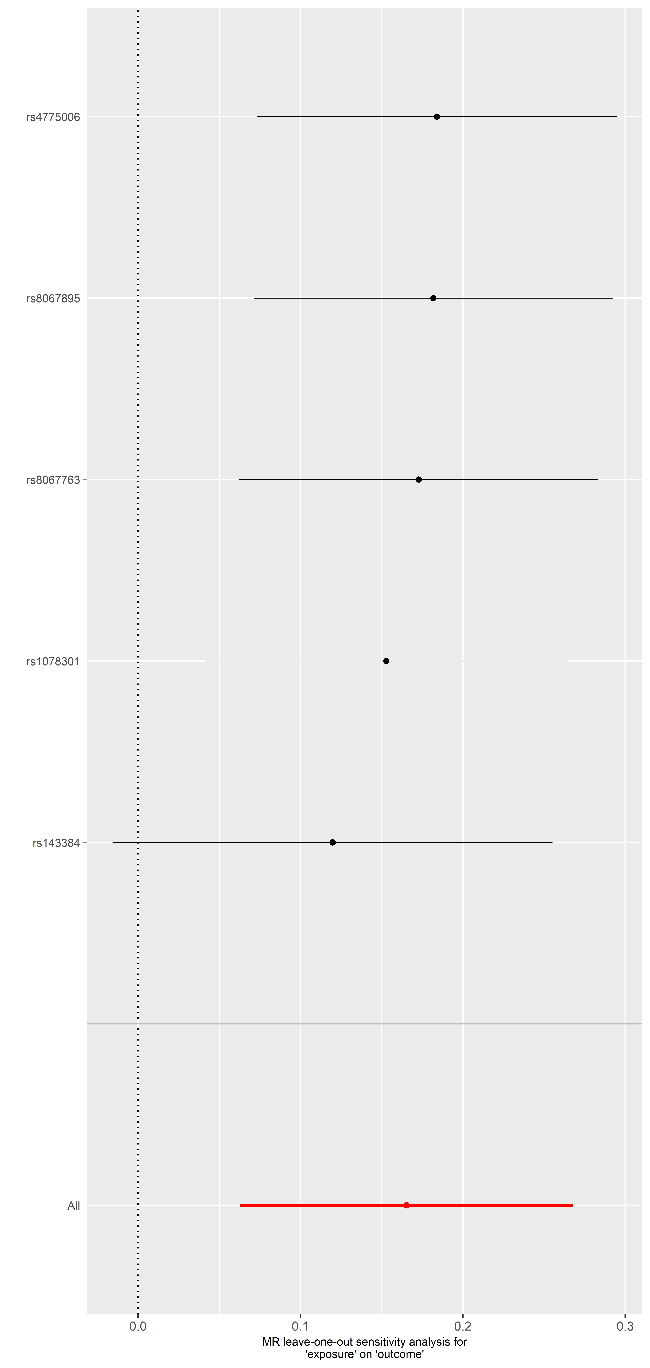


**A**

**B**

Supplementary figure1: Leave-one-out sensitivity analysis for the effect of knee osteoarthritis **(A)** and hip osteoarthritis **(B)** on the risk of type 2 diabetes.


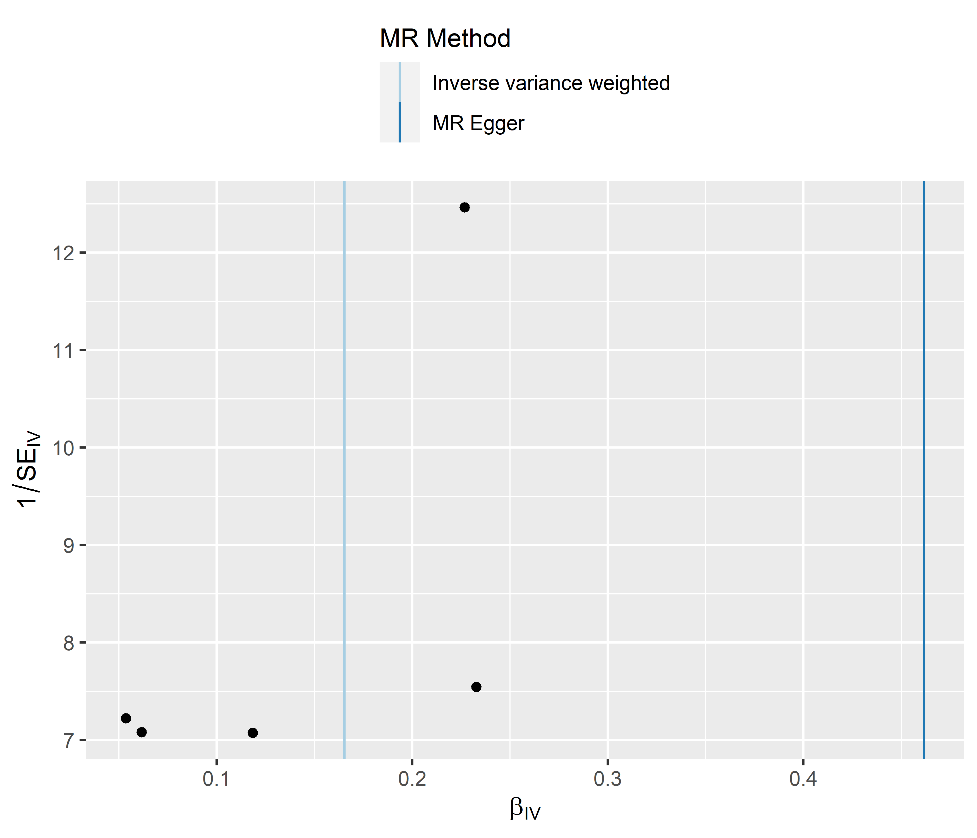

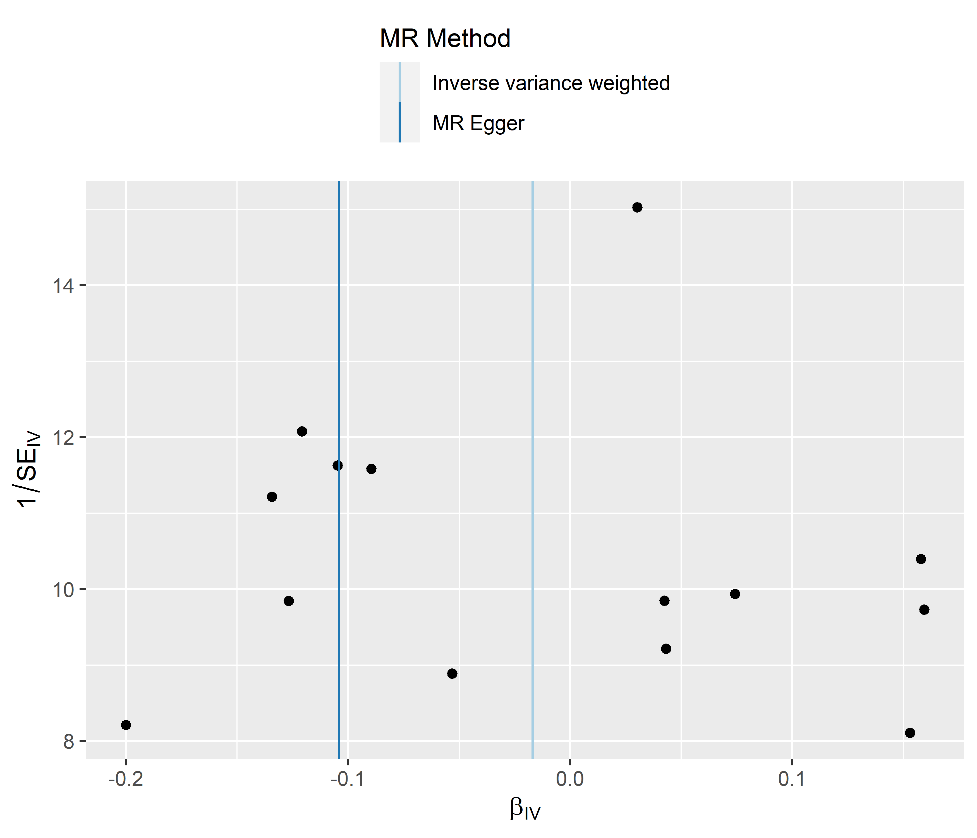


**A**

**B**

Supplementary figure2:funnel plot for the effect of knee osteoarthritis **(A)** and hip osteoarthritis **(B)** on the risk of type 2 diabetes.
