# Supplementary material for: Osteoarthritis and risk of type 2 diabetes: A two‐sample Mendelian randomization analysis
Source: J Diabetes. 2023 Jul 31;15(11):987–93. doi: 10.1111/1753-0407.13451 (PMC10667649; doi:10.1111/1753-0407.13451)
Supplement: Supplementary file 2 — Table S1. Characteristics of SNPs used in Mendelian randomization analysis of the effects of knee osteoarthritis (KOA) in type 2 diabetes (T2D). Table S2. Characteristics of SNPs used in Mendelian randomization analysis of the effects of hip osteoarthritis (HOA) in type 2 diabetes (T2D). Table S3. Two‐sample Mendelian randomized analyses for the associations of hip osteoarthritis with the risk of type 2 diabetes after adjusted by MR‐PRESSO. Table S4. Statistical power for the Mendelian randomization analysis. Table S5. List of genetic variants associated with more than one phenotype. [file JDB-15-987-s001.docx]

Supplementary Material

Supplementary File

Table S1. Characteristics of SNPs used in Mendelian randomization analysis of the effects of KOA in T2D.

Table S2. Characteristics of SNPs used in Mendelian randomization analysis of the effects of HOA in T2D

Table S3. Two-sample Mendelian randomized analyses for the associations of hip osteoarthritis with the risk of type 2 diabetes after adjusted by MRPRESSO

Table S4. Statistical power for the Mendelian randomization analysis.

Table S5. List of genetic variants associated with more than one phenotype.

Figure S1. The leave-one-out plot of the effects of KOA/HOA in T2D.

Figure S2. The funnel plot of the effects of KOA/HOA and in T2D.

Table S1. Characteristics of SNPs used in Mendelian randomization analysis of the effects of KOA in T2D.

Table S2. Characteristics of SNPs used in Mendelian randomization analysis of the effects of HOA in T2D

Table S3 Two-sample Mendelian randomized analyses for the associations of hip osteoarthritis with the risk of type 2 diabetes after adjusted by MRPROSS

Table S4 Statistical power for the Mendelian randomization analysis.

| **Exposure** | **Outcome** | **Variance** | **Power at different ORs and α=0.05** | | | |
| --- | --- | --- | --- | --- | --- | --- |
|  |  |  | **1.2** | **1.5** | **1.8** | **2.0** |
| KOA | T2D | 0.0006 | 0.22 | 0.83 | 1.00 | 1.00 |
| HOA | T2D | 0.0020 | 0.57 | 1.00 | 1.00 | 1.00 |
| **OR: odds ratio; KOA: knee osteoarthritis; HOA: hip osteoarthritis; T2D: type 2 diabetes** | | | | | | |

Table S5. List of genetic variants associated with more than one phenotype.

| SNPs | trait |  |
| --- | --- | --- |
| rs1078301 | Pain type experienced in last month: knee pain |  |
| rs143384 | Height in females, Height in males, Height, Hip circumference in males, Hip circumference, Hip circumference adjusted for BMI, Waist hip ratio in physically active indivdiuals, Waist hip ratio adjusted for BMI, Weight, Gene expression, Infant length, Joint mobility Beighton score, Arm fat-free mass left, Arm predicted mass left, Arm predicted mass right, Basal metabolic rate, Comparative height size at age 10, Forced expiratory volume in 1-second, Forced expiratory volume in 1-second, best measure, Forced expiratory volume in 1-second, predicted, Forced vital capacity, Forced vital capacity, best measure, Gonarthrosis, Hand grip strength left, Hand grip strength right, Impedance of leg left, Impedance of leg right, Impedance of whole body, Internal derangement of knee, Leg fat percentage left, Leg fat percentage right, Leg fat-free mass left, Leg fat-free mass right, Leg predicted mass left, Leg predicted mass right, Pain type experienced in last month: knee pain, Peak expiratory flow, Sitting height, Trunk fat mass, Trunk fat-free mass, Trunk predicted mass, Weight, Whole body fat mass, Whole body fat-free mass, Whole body water mass |  |
| rs4775006 | Pain type experienced in last month: knee pain |  |
| rs8067763 | Height, Comparative height size at 10 age |  |
| rs8067895 | Pain type experienced in last month: knee pain, Platelet count, Plateletcrit, White blood cell count, Heel bone mineral density, Heel bone mineral density left, Heel bone mineral density right, Impedance of leg left, Impedance of leg right, Impedance of whole body, Vascular or heart problems diagnosed by doctor: high blood pressure, Vascular or heart problems diagnosed by doctor: none of the above, Coronary artery disease |  |
| rs10492367 | Hip osteoarthritis, Osteoarthritis, Coxarthrosis, Height |  |
| rs10896015 | Mean corpuscular hemoglobin concentration, Platelet count, Plateletcrit, Red cell distribution width |  |
| rs11059094 | Arm fat mass left, Arm fat mass right, Arm fat percentage left, Arm fat percentage right, Arm fat-free mass left, Arm fat-free mass right, Arm predicted mass left, Arm predicted mass right, Basal metabolic rate, Body fat percentage, Body mass index, Comparative height size at age 10, Height, Hip circumference, Leg fat mass left, Leg fat mass right, Leg fat-free mass left, Leg fat-free mass right, Leg predicted mass left, Leg predicted mass right, Self-reported gout, Sitting height, Treatment with allopurinol, Trunk fat mass, Trunk fat percentage, Trunk fat-free mass, Trunk predicted mass, Waist circumference, Weight, Whole body fat mass, Whole body fat-free mass, Whole body water mass |  |
| rs11583641 | Sitting height |  |
| rs12040949 | Platelet count, Plateletcrit, Chronotype, Morning vs evening chronotype, Morning or evening person |  |
| rs12209223 | Height, Hip circumference adjusted for BMI, Waist circumference adjusted for physical activity, Arm fat-free mass left, Arm fat-free mass right, Arm predicted mass left, Arm predicted mass right, Basal metabolic rate, Comparative height size at age 10, Hip circumference, Sitting height, Trunk fat-free mas, Trunk predicted mass, Weight, Whole body fat-free mass, Whole body water mass |  |
| rs1913707 | Impedance of arm left |  |
| rs2396502 | Arm fat-free mass right, Arm predicted mass right, Heel bone mineral density, Heel bone mineral density left, Heel bone mineral density right, Height, Leg fat-free mass right, Leg predicted mass right, Sitting height, Trunk fat-free mass, Trunk predicted mass, Whole body fat-free mass, Whole body water mass |  |
| rs2785988 | Type II diabetes, Arm fat mass left, Arm fat mass right, Arm fat percentage left, Arm fat percentage right, Body fat percentage, Hip circumference, Impedance of arm left, Impedance of arm right, Impedance of whole body, Leg fat mass left, Leg fat mass right, Leg fat percentage left, Leg fat percentage right, Trunk fat mass, Trunk fat percentage, Umbilical hernia, Whole body fat mass |  |
| rs3774355 | Body mass index, High light scatter percentage of red cells, High light scatter reticulocyte count, Reticulocyte count, Reticulocyte fraction of red cells, Height, Comparative height size at age 10, Impedance of arm left, Impedance of arm right, Impedance of leg left, Impedance of leg right, Impedance of whole body |  |
| rs4338381 | Height, Height adults, Comparative height size at age 10, Sitting height |  |
| rs7571789 | Hand grip strength left, Hand grip strength right, Self-reported osteoarthritis |  |
|  |  |  |
|  |  |  |
